# Primary Ciliary Dyskinesia Due to Microtubular Defects is Associated with Worse Lung Clearance Index

**DOI:** 10.1007/s00408-018-0086-x

**Published:** 2018-01-24

**Authors:** S. Irving, M. Dixon, M. R. Fassad, E. Frost, J. Hayward, K. Kilpin, S. Ollosson, A. Onoufriadis, M. P. Patel, J. Scully, S. B. Carr, H. M. Mitchison, M. R. Loebinger, C. Hogg, A. Shoemark, A. Bush

**Affiliations:** 1 National Heart and Lung Institute, Imperial College London, London, UK; 2 Royal Brompton & Harefield NHS Trust, Sydney Street, London, SW3 6NP UK; 3 Genetics and Genomic Medicine, University College London (UCL) Great Ormond Street Institute of Child Health, London, UK; 4 Human Genetics Department, Medical Research Institute, Alexandria University, Alexandria, Egypt; 5 Regional Molecular Genetics Laboratory, Great Ormond Street Hospital for Children NHS Foundation Trust, London, UK; 6 Division of Genetics and Molecular Medicine, Department of Medical and Molecular Genetics, King’s College London School of Medicine, Guy’s Hospital, London, UK; 7 Division of Molecular and Clinical Medicine, University of Dundee, Dundee, UK

**Keywords:** Lung function, Paediatrics, Rare disease, Ciliopathy

## Abstract

**Purpose:**

Primary ciliary dyskinesia (PCD) is characterised by repeated upper and lower respiratory tract infections, neutrophilic airway inflammation and obstructive airway disease. Different ultrastructural ciliary defects may affect lung function decline to different degrees. Lung clearance index (LCI) is a marker of ventilation inhomogeneity that is raised in some but not all patients with PCD. We hypothesised that PCD patients with microtubular defects would have worse (higher) LCI than other PCD patients.

**Methods:**

Spirometry and LCI were measured in 69 stable patients with PCD. Age at testing, age at diagnosis, ethnicity, ciliary ultrastructure, genetic screening result and any growth of *Pseudomonas aeruginosa* was recorded.

**Results:**

Lung clearance index was more abnormal in PCD patients with microtubular defects (median 10.24) than those with dynein arm defects (median 8.3, *p* = 0.004) or normal ultrastructure (median 7.63, *p* = 0.0004). Age is correlated with LCI, with older patients having worse LCI values (*p* = 0.03, *r* = 0.3).

**Conclusion:**

This study shows that cilia microtubular defects are associated with worse LCI in PCD than dynein arm defects or normal ultrastructure. The patient’s age at testing is also associated with a higher LCI. Patients at greater risk of obstructive lung disease should be considered for more aggressive management. Differences between patient groups may potentially open avenues for novel treatments.

**Electronic supplementary material:**

The online version of this article (10.1007/s00408-018-0086-x) contains supplementary material, which is available to authorized users.

## Introduction

**Fig. 1:**
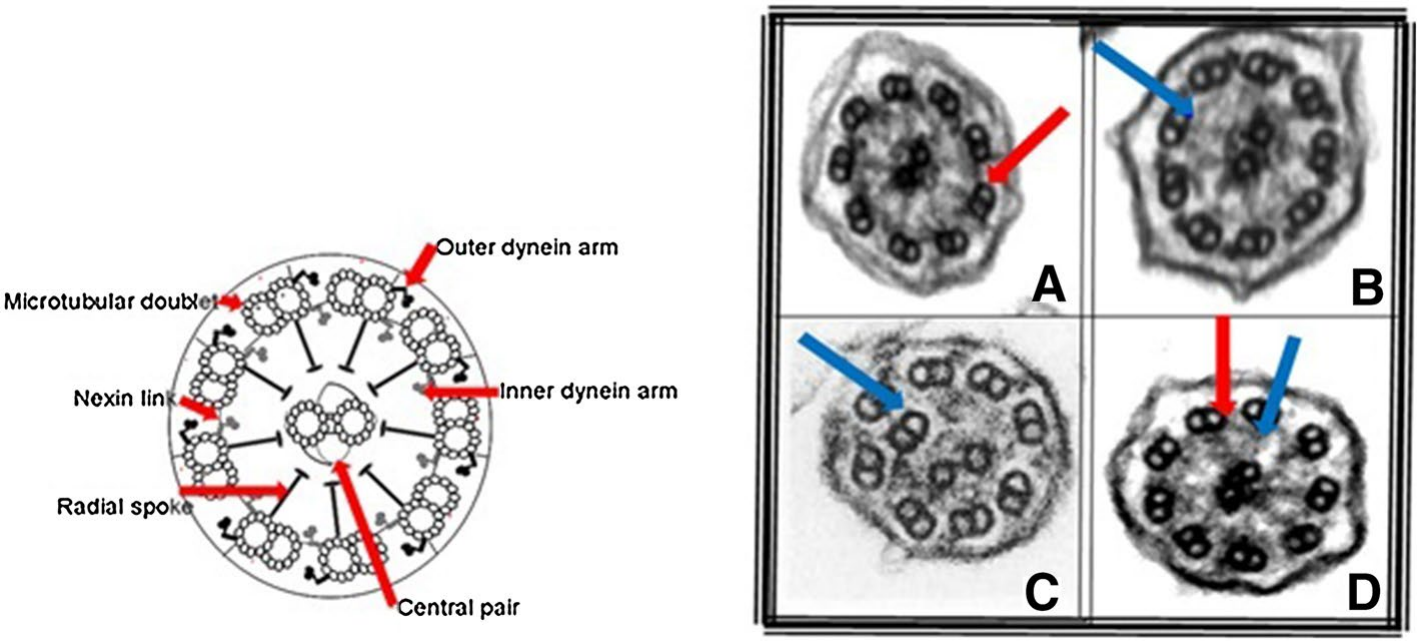
A diagram of a normal cilium on the left, with a normal ultrastructure, showing microtubular doublets, a central pair, nexin links and radial spokes and inner and outer dynein arms. The electron micrographs on the right show common PCD defects, in *A*, absence of the outer dynein arms, in *B*, absence of inner dynein arms, in *C*, absence of inner dynein arms and microtubular disorganisation and in *D*, absence of both the inner and outer dynein arms

Primary ciliary dyskinesia (PCD) is an inherited condition characterised by repeated upper and lower respiratory tract infections and chronic secretory otitis media. Approximately 50% patients have disorders of laterality. Morbidity is significant with most patients developing obstructive lung disease and bronchiectasis [1–4]. Diagnosis is complex, and may include combinations of suggestive clinical features, low nasal nitric oxide and abnormal ciliary waveform on high-speed videomicroscopy, abnormal ultrastructure defect on electron microscopy (EM) and/or positive genetic tests [5]. There is no uniformly applicable gold standard. Normal ciliary ultrastructure and common PCD ultrastructural defects are shown in Fig. 1.

Ciliary ultrastructural abnormalities can be divided into three broad classes; defects affecting the dynein arms, defects of the microtubules and PCD with normal ultrastructure. The majority of defects occur in the dynein motor proteins which drive ciliary beating, known as the inner and outer dynein arms (IDA, ODA, respectively); these may be caused by gene mutations either leading to failure of synthesis of proteins which form the structural components of the dynein arm proteins or proteins involved in their assembly [1]. Defects of the microtubular arrangement include absence of the central microtubular pair together with transposition of a peripheral doublet (usually caused by defects in radial spoke head genes); and microtubular disorganisation, also often with loss of the inner dynein arm, which usually results from mutations in the ‘molecular ruler’ and dynein regulatory complex proteins [6, 7]. 15–30% of patients with PCD will have no demonstrable ultrastructural defect on TEM, including those carrying mutations in genes encoding specific ODA (DNAH11) and central pair (HYDIN) proteins [8]. In these patients, confirmation of the diagnosis is made by identification of biallelic mutations in a PCD disease-causing gene or deemed ‘highly likely’ based on persistent diagnostic ciliary waveform abnormalities on high-speed videomicroscopy which may be combined with suggestive clinical features and low nasal nitric oxide [9].

Primary ciliary dyskinesia is a heterogeneous condition, with wide variability in clinical status, lung function and outcomes. Previous studies using spirometry, the main tool for accessing disease severity in terms of lung function [specifically forced expiratory volume in 1 s (FEV_1_)], have been conflicting [10–12]. A large study recently found spirometry is worse in patients with microtubular defects than in patients with ODA ± IDA defects, although not all investigators have reported this [13–17].

Lung clearance index (LCI) is derived from multi-breath washout of nitrogen or an exogenous inert gas and is a sensitive measure of distal airway function [18]. The normal range is broadly similar from school age to adulthood. LCI has been shown to be more sensitive to early airways disease than spirometry in cystic fibrosis [19], and is abnormal in patients with PCD [17, 20]. It may be that this more sensitive parameter is a better tool to access lung function differences in PCD than spirometry.

We hypothesised that LCI would vary between PCD patients with different ultrastructural defects, with the worst results in those with microtubular defects.

## Materials and Methods

### Ethics

The clinical study was approved by NRES Committee South East Coast—Brighton & Sussex (REC reference: 10/H1101/69). Genetic analysis was approved by NRES Committee London Bloomsbury for UCL Great Ormond Street Institute of Child Health/Great Ormond Street Hospital (REC reference: 08/H0713/82).

### Recruitment

PCD patients with normal ultrastructure, microtubular defects or outer dynein arm with or without inner dynein arm (ODA ± IDA) were recruited.

Initial recruitment was conducted across the paediatric and adult age range, and 74 paediatric and adult PCD patients willing to take part were recruited from the respiratory clinics at the Royal Brompton Hospital. However, five patients over 50 years of age originally recruited have been excluded from the main dataset. These patients were all of white ethnicity, 4/5 had an ODA ± IDA defect (1 had microtubular defect) and we were unable to recruit equivalent patients of other ethnic origin, or with defects other than ODA ± IDA, in this age group. For this reason, they were removed from the main analysis due to introducing an unacceptable risk of bias into the data. Demographics of these removed patients are available in the OLS.

The remaining 69 patients are shown in Table 1. All had undergone detailed evaluation including history and clinical examination including a nasal ciliary brush biopsy which was sent for light microscopic examination and ultrastructural examination by EM.

**Table 1:** Patient demographics and lung function results

Group	Number	Age (years) [median, (range)]	Age at EM diagnosis (years) (median, (range))	BMI [median, (range)]	Gender (M/F)	*Pseudomonas aeruginosa* isolated (%)	Ethnicity (White/South Asian)	Nasal nitric oxide (nl/min) [median, (range)]	FEV_1_* z*-score [median, (range)]	FEF_25–75%_* z*-score [median, (range)]	LCI [median, (range)]
Total	69	13 (4–41)	6 (0–37)	19.4 (10.7–27.5)	25/44	30	31/36	14 (0^c^–159)	− 1.98 (− 5.33 to 0.73)	− 2.17 (− 5.78 to 0.55)	8.44 (5.84–14.98)
Normal ultrastructure	14	13 (7–18)	6 (0–15)	18.9 (13.2–24.0)	6/8	21	6/7^a^	12 (2–136)	− 1.26 (− 3.68 to 0.73)	− 1.19 (− 3.22 to 0.0.1)	7.63 (6.2 to 11.82)
ODA ± IDA	39	14 (4–32)	10 (0–28)	20.8 (12.9–27.5)	17/22	30	17/22	14 (0^c^–30)	− 2.03 (− 4.81 to 0.32)	− 2.2 (− 4.85 to 0.55)	8.3 (5.84–14.98)
Microtubular defects	16	13 (4–41)	7 (0–37)	18.7 (10.7–25.9)	2/14	19	8/7^b^	16 (2–159)	− 3.1 (− 5.33 to − 0.36)	− 2.33 (− 5.78 to − 0.67)	10.24 (7.97–14.1)
*Statistical significance*		*NS*	*NS*	*NS*				*NS*	*p* = *0.04*^d^	*p* = *0.03*^e^	*p* = *0.0013*^f^

LCI, FEF_25–75%_ and FEV_1_ were different between groups. There was no difference in age at testing, age at EM diagnosis, nasal nitric oxide or BMI between the groups. There were more females than males in this cohort. 30% of patients overall grew *Pseudomonas aeruginosa*

^a^group contains 1 mixed race patient

^b^group contains 1 black patient

^c^Below lower limit of detection

^d^Kruskal–Wallis test for non-parametric group analysis *p* = 0.04, Mann–Whitney tests between groups show difference between microtubular defects and normal ultrastructure groups only (*p* = 0.02). There is no significant difference between microtubular defects and ODA ± IDA groups, or normal ultrastructure and ODA ± IDA groups

^e^Kruskal–Wallis test for non-parametric group analysis *p* = 0.03, Mann–Whitney tests between groups show difference between microtubular defects and normal ultrastructure groups only (*p* = 0.02). There is no significant difference between microtubular defects and ODA ± IDA groups, or normal ultrastructure and ODA ± IDA groups

^f^Kruskal–Wallis test for non-parametric group analysis *p* = 0.0013, Mann–Whitney tests between groups show difference between microtubular defects and normal ultrastructure groups (*p* = 0.0004), and difference between microtubular defects and ODA ± IDA groups (*p* = 0.04),but no difference between normal ultrastructure and ODA ± IDA groups

Inclusion criteria were a positive or highly likely diagnosis of PCD according to current European guidelines [9] based on:

1. A hallmark TEM defect.
2. Biallelic changes in a known PCD gene.
3. Consistent reproducible defects on high-speed video microscopy, coupled with persistently low nasal NO and clinical features of PCD.

The date on which the EM sample was received was used as a surrogate for age at diagnosis (full details of diagnostic process are given below). Patients were stable at time of recruitment with no increase in symptoms in the 2 weeks prior to testing, and no new pathogens grown on cough swab or sputum sample. Note was made if the patient had ever grown *Pseudomonas aeruginosa*. As PCD is a rare disease, all eligible patients were approached and no statistical sampling method was used.

### Lung Function

Patients performed spirometry (ATS/ERS guidelines [21]), from which forced expiratory volume in 1 s (FEV_1_) and forced expiratory flow 25–75% of vital capacity (FEF_25–75%_)* z*-scores were calculated using the global lungs initiative (GLI) [22]. At the same visit, after spirometry, they performed multiple breath washout test (MBW) from which LCI was calculated. A sulphur hexafluoride (SF_6_) washout with a photoacoustic gas analyser (Innocor, Innovision, Denmark) was used, with an open breathing circuit, as described previously, with a minimum of 2 runs of acceptable quality, in accordance with ERS/ATS guidelines [18, 20, 23]. Abnormal LCI was defined as > 7.4 [18, 20].

### Electron Microscopy

Samples were fixed in 2.5% glutaraldehyde in cacodylate buffer, processed for electron microscopy (EM) and defects were quantified [7]. Briefly, cells were washed in sodium cacodylate buffer, post-fixed with 1% osmium tetroxide and centrifuged in 2% agar to generate a pellet. Using a series of increasing concentrations of methanol followed by propylene oxide, cells were dehydrated before being embedded in resin. 70–90 nm sections were cut using a Reichert Ultracut-E ultramicrotome and mounted onto copper grids. Heavy metal staining used uranyl acetate and lead citrate. Assessment of the respiratory epithelium and ciliary ultrastructure were made on a transmission electron microscope. Quantification of cells, microtubular arrangement in the axoneme and presence of dynein arms was performed by a clinical electron microscopist. 100–300 cilia were analysed in transverse section and note of ciliary orientation and cell health and appearance of longitudinal ciliary sections was made before a diagnosis of PCD confirmed. Care was taken to assess cilia from a number of healthy cells from locations proximal and distal to the epithelial cell surface.

### Genetics

Mutation data were generated using next-generation sequencing methods, either whole exome sequencing or screening of custom targeted gene panels employing TruSeq (Illumina Inc) and SureSelect (Agilent Technologies) systems and an illumina sequencing system with variant calling performed as previously described [24, 25].

### Statistical Methods

Sample size was opportunistic, since there are no data to inform a power calculation. Non-parametric statistics were used for lung function results. Differences between three and more groups were assessed by Kruskal–Wallis test; and then pairwise between two groups by Mann–Whitney test. Spearman’s *r* was used for correlation.

SPSS (IBM, NY, USA) was used for statistical analysis; graphs were plotted using GraphPad Prism (GraphPad Software Inc, CA, USA).

## Results

### Ciliary Ultrastructure

**Fig. 2:**
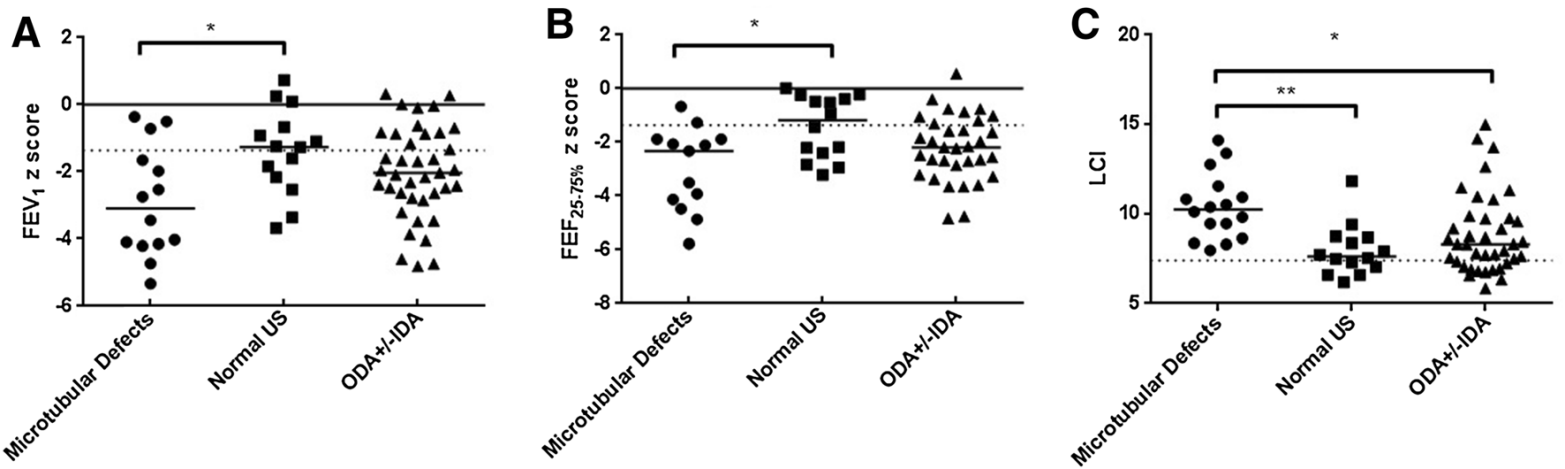
Differences in lung function tests between groups. Those with normal ultrastructure had the best results, and those with microtubular defects the worst, with ODA ± IDA intermediate. Panel A FEV_1_ *p = 0.02; Panel B FEF_25–75%_ *p = 0.02; Panel C LCI *p = 0.04 **p = 0.0004

Demographics are given in Table 1. There was no significant difference between the three groups in age at testing, age at diagnosis, BMI or nasal nitric oxide. Median LCI, FEV_1_ and FEF_25–75%_ were abnormal in all three groups (Fig. 2). The microtubular defects group all had abnormal LCI, but not FEV_1_ or FEF_25–75%_. Genotyping was available for 55 out of the 69 PCD patients (Table 2).

**Table 2:** Ultrastructure defect groupings, including genetic results where available

Group		Number	Genetic result
ODA ± IDA	ODA	39	*9 DNAH5, 1 ARMC4, 1 LRRC6, 1 DNAAF3, 2 DNAI1, 1* pathogenic mutations in a known PCD gene were not found, 9 no sample taken
		24	
	ODA + IDA	15	*5 DNAAF3, 1 DYX1C1, 1 ZMYND10, 1 CCDC103, 4 LRRC6,, 1 SPAG1, 2* no sample taken
Normal ultrastructure		14	*6 DNAH11, 3 HYDIN, 1 CCDC103, 1 RPGR*
			1 no sample taken, 2 pathogenic mutations in a known PCD gene were not found*
Microtubular defects	Central complex	16	*2 RSPH4A, 2* no sample taken
		4	
	Microtubular defects + IDA	12	*5 CCDC39, 4 CCDC40, 1* pathogenic mutations in a known PCD gene *were not found, 2 no sample taken*

*Three patients with no sample taken for genetics or no gene found showed consistent reproducible abnormalities on high-speed video microscopy

On non-parametric statistical analysis, LCI was significantly worse in patients with microtubular defects than the other two groups (Table 1; Fig. 2, Kruskal–Wallis result for three groups *p* = 0.0013, pairwise Mann–Whitney result for microtubular defects versus ODA ± IDA *p* = 0.004, and microtubular defects vs normal ultrastructure *p* = 0.0004). FEV_1_ and FEF_25–75%_ were also significantly worse in patients with microtubular defects compared with the group with normal ultrastructure (Table 1; Fig. 2, Kruskal–Wallis result for three groups *p* = 0.04 for FEV_1_* z*-score and *p* = 0.03 for FEF_25–75%_, pairwise Mann–Whitney result for normal ultrastructure versus microtubular defects *p* = 0.02 for both, no other significant differences).

### Current Age and Age at Diagnosis

**Fig. 3:**
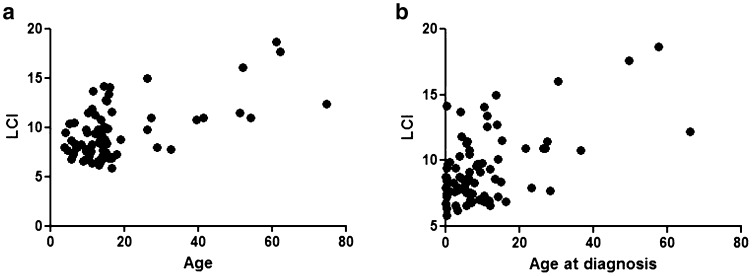
Relationship between age (**a**) and age at diagnosis (**b**) and LCI. Age and LCI correlate significantly (*p* = 0.0012, *r* = 0.4), as does age and FEV_1_* z*-score (*p* = 0.004, *r* = 0.3)

Current age (at time of testing) was correlated with LCI and FEV_1_ (Fig. 3; age and LCI *p* = 0.04 *r* = 0.3, age and FEV_1_ *p* = 0.008, *r* = − 0.3), but not FEF_25–75%_. LCI was not significantly correlated with age at diagnosis, whereas FEV_1_ was inversely correlated (*p* = 0.02, *r* = − 0.4). It was possible that younger patients were diagnosed earlier, as awareness of PCD has improved, thus introducing a bias, but there was no change in age at diagnosis over time (results in OLS).

### Ethnicity

Ethnic groups were equally distributed between EM groups (Table 1) and there is no statistically significant difference between LCI, FEV_1_, FEF_25–75%_, nasal nitric oxide, age or age at diagnosis between them.

### Ciliary Function

45 patients had predominantly static cilia on light microscopy, and 21 had predominantly dyskinetic. There was no difference in LCI, FEV_1_ and FEF_25–75%_ between these groups (data in OLS). Where beat frequency was measurable with non-static cilia, there was no relationship between beat frequency and lung function (see OLS for full data). There was no relationship between nasal nitric oxide results and lung function (see OLS).

## Discussion

We have shown that PCD patients with microtubular defects have worse LCI compared to groups with normal ultrastructure and dynein arm defects. Microtubular defects were first linked to comparatively worse lung function and decline in previous spirometry-based studies [13, 16], but these differences in LCI are novel, showing the utility of this test as a sensitive tool to investigate lung function differences in PCD. This study supports the hypothesis that there are potential outcome differences between different ultrastructural groups.

Unlike in previous work, there was no significant difference in FEV_1_ and FEF_25–75%_ between ODA ± IDA and microtubular defects [13]. This may be because the previous article used percent predicted, whereas we have used* z*-scores. Furthermore, the Wilcoxon signed-rank test for comparison between groups was used, whilst here we used a more stringent approach [16]. However, more data are needed to understand this discrepancy.

The mechanism by which different ultrastructural defects may give rise to differences in lung function in vivo is not clear. Ciliary motility differs between defects which may affect mucociliary transport, but why microtubular defect patients with some ciliary motility (such as the circling pattern seen in transposition defects) should have worse lung function than ODA ± IDA patients with completely static cilia is not known. Cilia also have a role in cell signalling, and we speculate that this could differ in different ultrastructural groups. In our centre, treatment decisions are based on clinical assessment rather than ultrastructure defect, so it is unlikely any differences arise from treatment differences.

One weakness of this study is the small cohort size. Until very recently, there was no commercially available validated LCI equipment, and measurements were restricted to tertiary centres, and so very large multicentre cohort studies (such as those published in spirometry) were not possible. Recent advances in equipment mean this will change in the coming years, and so establishing the role of this test in these patients is important.

Green et al. [26] found no significant correlation between age or age at diagnosis and lung function in their 2012 PCD cohort. In our cohort we found a correlation between age and LCI and FEV1. This could have been due to a number of factors. Our cohort is larger so may have been better powered to detect this difference, and also Green et al’s cohort contained no patients over the age of 18 years, so the effect may be less pronounced in paediatric patients. In this study, we have used age at EM diagnosis as a surrogate measure for age at diagnosis, due to incomplete and varied history of our patients prior to their referral to the PCD centre. This is an acknowledged compromise and may be why no significant association was found.

As many patients do not solely attend our clinic, the microbiological results are incomplete. However, the prevalence of *Pseudomonas aeruginosa* seen here (30%) is similar to previous reports; a large multicentre study gives a prevalence of 37% overall [11], a large adult patient cohort 45% [16] and another showed a varying prevalence in different calendar years of between 15 and 47% [27].

In our study, median BMI for all ultrastructure groups was in the healthy range (Table 1). This differs from the previous study [13], where microtubular defects patients had a lower BMI than the other groups. The exact reason for this discrepancy is unclear and may relate to differences in management protocols between centres, but excludes the possibility that low BMI was the cause of poorer lung function results in our microtubular defects patients.

In summary, in PCD, microtubular defects ultrastructure group and older age are associated with worse LCI. We cannot explain this intriguing finding, and more work is needed to address the pathways whereby different ultrastructure affects patient outcomes. In the future, determining the reasons for these differences could open up new treatment pathways, and delineation of high risk groups may allow more targeted intensive treatment to improve prognosis.

## Electronic Supplementary material

Below is the link to the electronic supplementary material.

Supplementary material 1 (DOCX 102 KB)
