# Supplementary material for: Primary Ciliary Dyskinesia Due to Microtubular Defects is Associated with Worse Lung Clearance Index
Source: Lung. 2018 Jan 24;196(2):231–8. doi: 10.1007/s00408-018-0086-x (PMC5854730; doi:10.1007/s00408-018-0086-x)
Supplement: Supplementary file 1 — Supplementary material 1 (DOCX 102 KB) [file 408_2018_86_MOESM1_ESM.docx]

**Additional methods**

**Healthy control data**

47 healthy volunteers were recruited. The mean age of volunteers was 21.6 years (range 3.2-51.3), 20 patients were children and young people under the age of 16, and 24 were adults. There were 22 males, mean height was 148cm (range 88cm to 183cm) weight was 50.7kg (range of 15.3kg to 93.6kg).

41 of 44 subjects completed three washouts, and three completed two washouts. The three who did not perform the full three washouts were all children who declined to continue after the second washout. Six subjects who completed three washouts had one repeat discarded, either due to poor quality of the trace (due to cough, leaks, or non-tidal breathing pattern) (n=4) or an FRC that fell outside the 10% difference from the other washouts thus suggesting it was technically unacceptable (n=2). No subjects reported any adverse effects from the MBW, other than a dry mouth, probably resulting from the dryness of the test gas. LCI was normally distributed in the healthy control group, so parametric statistics could be used.

The mean (standard deviation) of LCI in this healthy control group was 6.6 (0.68), range 5.51 to 8.35. The 8.35 result appears visually to be an outlier as the next lowest result is 7.61.

**Additional results**

Growth of *Pseudomonas Aeruginosa* was not associated with worse LCI, FEV_1_ or FEF_25-75%_ in these patients. Positive/negative for *Pseudomonas aeruginosa* groups did not have significantly different lung function results (Figure e1) LCI p=0.3, FEV_1_ p=0.6, FEF_25-75%_ p=0.2.


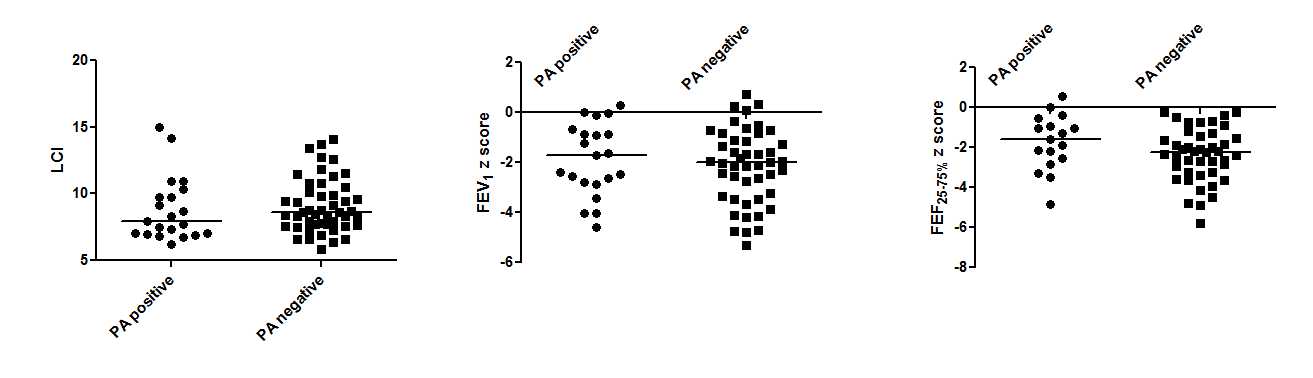


***Figure e1 – No significant difference between LCI (p=0.3), FEV_1_ (p=0.6) or FEF_25-75%_ (p=0.2) in groups that had or had not grown Pseudomas Aeruginosa***

There was no change in age at diagnosis over time i.e. younger patients were not diagnosed earlier, potentially introducing a bias (Figure e2).


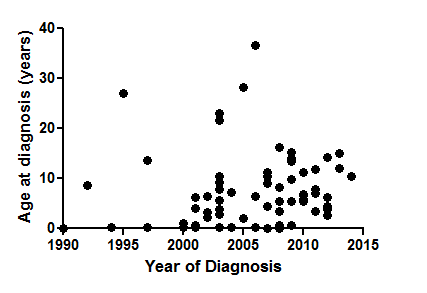


***Figure e2 – there is no significant relationship between year of diagnosis and age at diagnosis***

Beat frequency was measurable in 19 patients, there was no relationship between beat frequency and lung function (Figure S3, LCI p=0.09, FEV1 p=0.4, FEF25-75% p=0.4 (Spearman’s R)).


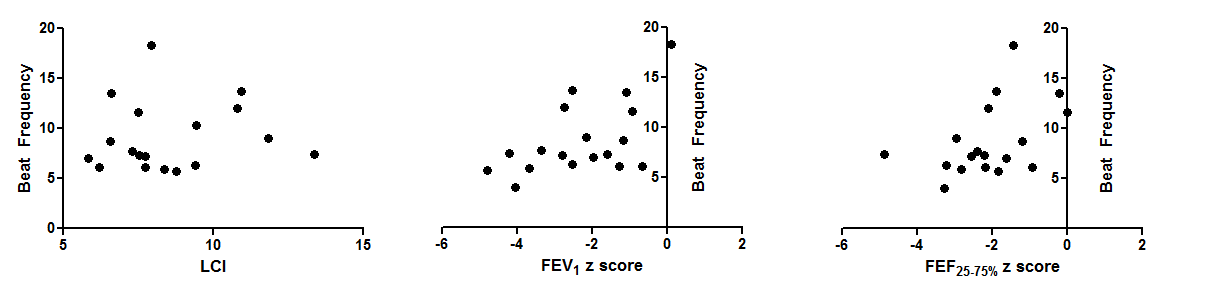


***Figure e3 – Lung function with beat frequency, there is no significant correlation***

66 patients had abnormalities on light microscopy, of which 45 patients had totally or partially static cilia and 21 patients had dyskinetic cilia. The remaining 3 patients in the main data set were excluded from this analysis as they had light microscopy carried out before 2006, using a different method (a photodiode measurement of CBF using a 40x objective compared to high speed video assessment with a 100x objective). There was no difference in LCI, FEV1 and FEF25-75% between these groups (Figure e4, LCI p=0.3, FEV_1_ p=0.09 and FEF_25-75%_ p=0.9 respectively (Mann-Whitney)).


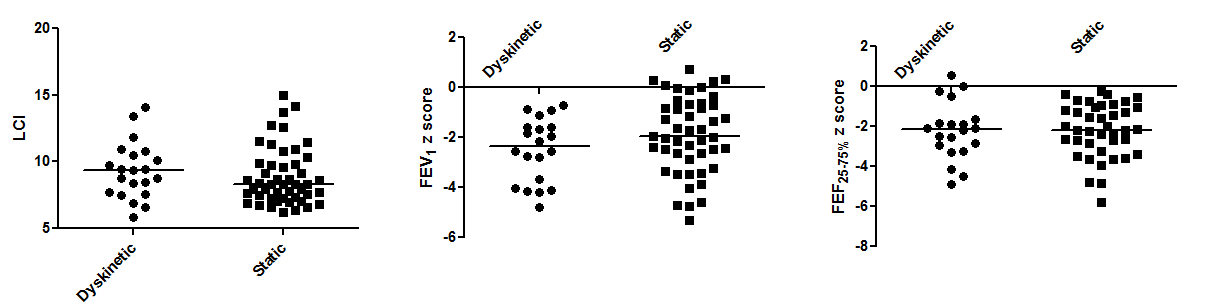


***Figure e4 – Lung function results by light microscopy groups, there was no significant difference***

Those patients with nasal nitric oxide results had these compared to LCI and spirometry parameters (Figure e5). There were no correlations seen.

***
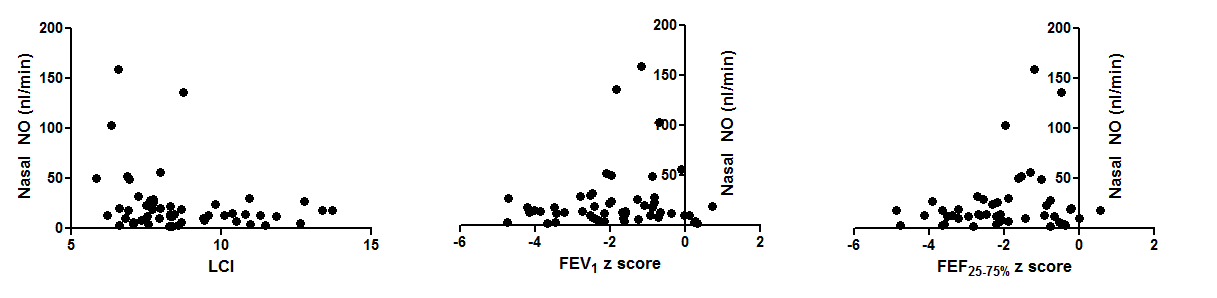
Figure e5 – Nasal nitric oxide (NNo) compared to LCI, FEV_1_ z score and FEF_25-75%_ z score. There were no statistically significant correlations***

**Comparison of study cohort to full PCD cohort**

The demographics and available clinical information for the cohort recruited compared with the population of PCD patients in our adult and paediatric respiratory clinics is shown below.

|  | FEV1 (% predicted) | Age | BMI | Defect | Ethnicity | PA growth |
| --- | --- | --- | --- | --- | --- | --- |
| Paediatric total cohort  n=117 | 78% | 12 | 18 | ODA+/-IDA 65%  MTD 18%  Normal 18% | Caucasian 39%  South Asian 56%  Other 5% | Not known |
| Paediatric study cohort n=57 | 75% | 11 | 18 | ODA+/-IDA 55%  MTD 20%  Normal 25% | Caucasian 39%  South Asian 58%  Other 3% | 25% |
| Adult total cohort  n=151 | 71% | 35 | Not known | ODA+/-IDA 66%  MTD 20%  Normal 14% | Not known | 68% |
| Adult study cohort  n=13 | 67% | 25 | 22 | ODA+/-IDA 62%  MTD 31%  Normal 7% | Caucasian 69%  South Asian 31%  Other 0% | 54% |

***Table e1 – Demographics of study population compared with available adult and paediatric PCD populations.***

There are some missing data in the available clinical database. BMI and ethnicity is not currently recorded for adult patients on clinical data. Many PCD patients have shared care arrangements with local hospitals, and so full microbiology data is not available for the majority of patients to confirm growth of P. aeruginosa.

In general, there is a good agreement between the study groups and total populations, although the adult recruited cohort is small.

Our cohort are also comparable to previously published data. 140 patients from our centre were included in the cross sectional and longitudinal analysis of lung function and clinical characteristics in multiple centres conducted by Maglione et al (Pediatr Pulmonol. 2014 Dec;49(12):1243-50). In that study, FEV1 was similar (-1.37 z scores versus -1.98 z scores), percentage with ODA+/-IDA defects (64% versus 56%) and infection with Pseudomonas Aeruginosa (36% versus 30%).

**Additional analysis details**

Age does not differ between the ultrastructure defect categories (Kruskall-Wallis p=0.75), but is weakly associated with LCI (Spearman’s rank p=0.04, r=0.3). For that reason, it would be desirable to attempt a multivariate analysis to rule out any effects of age on the differences seen in LCI between ultrastructure groups, and professional statistical advice was sought.

In these patients, both LCI and age are non-parametrically distributed. LCI can be transformed using a natural logarithm to a parametric continuous variable, but age remains skewed when transformed. For this reason, the most appropriate data handling method was to break age down to categorical variables of paediatric (<17 years of age) and adult age groups.

When a linear regression is performed, there is no significant correlation between these age variables and LCI, and therefore further modelling to a multivariate analysis with ultrastructure group is not appropriate in these data, and univariate analysis as presented in the main paper remains the most appropriate method of performing this analysis.
